# Site-Specific Ser/Thr/Tyr Phosphoproteome of *Sinorhizobium meliloti* at Stationary Phase

**DOI:** 10.1371/journal.pone.0139143

**Published:** 2015-09-24

**Authors:** Tao Liu, Chang Fu Tian, Wen Xin Chen

**Affiliations:** 1 State Key Laboratory of Agrobiotechnology, and College of Biological Sciences, China Agricultural University, Beijing, China; 2 Key Laboratory of Soil Microbiology, Ministry of Agriculture, China Agricultural University, Beijing, China; 3 Rhizobium Research Center, China Agricultural University, Beijing, China; Universität Regensburg, GERMANY

## Abstract

*Sinorhizobium meliloti*, a facultative microsymbiont of alfalfa, should fine-tune its cellular processes to live saprophytically in soils characterized with limited nutrients and diverse stresses. In this study, TiO_2_ enrichment and LC-MS/MS were used to uncover the site-specific Ser/Thr/Tyr phosphoproteome of *S*. *meliloti* in minimum medium at stationary phase. There are a total of 96 unique phosphorylated sites, with a Ser/Thr/Tyr distribution of 63:28:5, in 77 proteins. Phosphoproteins identified in *S*. *meliloti* showed a wide distribution pattern regarding to functional categories, such as replication, transcription, translation, posttranslational modification, transport and metabolism of amino acids, carbohydrate, inorganic ion, succinoglycan etc. Ser/Thr/Tyr phosphosites identified within the conserved motif in proteins of key cellular function indicate a crucial role of phosphorylation in modulating cellular physiology. Moreover, phosphorylation in proteins involved in processes related to rhizobial adaptation was also discussed, such as those identified in SMa0114 and PhaP2 (polyhydroxybutyrate synthesis), ActR (pH stress and microaerobic adaption), SupA (potassium stress), chaperonin GroEL2 (viability and potentially symbiosis), and ExoP (succinoglycan synthesis and secretion). These Ser/Thr/Tyr phosphosites identified herein would be helpful for our further investigation and understanding of the role of phosphorylation in rhizobial physiology.

## Introduction

Rhizobia live saprophytically in soil and occasionally form nitrogen-fixing nodules with legumes. In this lifecycle, rhizobia need to adapt to conditions both within plant and in soils. The available limited nutrients not only fluctuate in soils with periods of weeks or months, but also show significant differences in concentration between the plant cell environment and in the soils. To survive in these conditions, rhizobia should fine-tune the regulation machinery to respond competitively in the community [1, 2].

The phosphoryl group resulted from a phosphorylation event may subtly alter a protein’s functional properties due to its intrinsic biophysical properties such as high charge density [3]. In rhizobia, the role of phosphorylation in regulating cellular physiology is mainly presented in two-component regulatory system such as RegS-RegR and FixL-FixJ in microaerobic adaption and nitrogen fixation [4–8], ExoS-ChvI in succinoglycan and flagellum production [9, 10], PhoR-PhoB in phosphate utilization [11, 12], DctB-DctD in dicarboxylate transport [13], SMa0113-SMa0114 in catabolite repression [14], ActS-ActR in adaption to pH stress and microaerobic condition etc. [15, 16]. In a typical two-component system, the histidine kinase activates the response regulator by phosphorylation, which in turn controls the transcription of target genes. This kind of two-component kinases (His/Asp phosphorylation) together with Hanks type kinases (Ser/Thr phosphorylation), BY kinases (bacterial tyrosine kinases, Tyr phosphorylation) and phosphotransferase system (His phosphorylation) are the four major families of kinases managing bacterial phosphorylation processes upon sensing various signals [17]. The carbohydrate and nitrogen phosphotransferase systems have also been studied and demonstrated as regulators of diverse processes in rhizobia [18–20]. ExoP of *S*. *meliloti* is a protein tyrosine kinase involved in succinoglycan synthesis and secretion [21, 22]. However, a global view of phosphoproteome of rhizobia remains unknown.

Recent development in methodology of global phosphoproteomics has inspired site-specific analysis of phosphoproteomes in around 20 bacterial species such as *B*. *subtilis* [23], *E*. *coli* [24], *Klebsiella pneumoniae* [25], *Mycobacterium tuberculosis* [26], *Listeria monocytogenes* [27], *Cyanobacterium synechococcus* [28], though restricted to Ser/Thr/Tyr protein phosphorylation due to chemical instability of His/Asp phosphorylation at lower pH values (pH < 8) [29]. The available bacterial phosphoproteomes vary significantly among species in terms of sizes and structures, such as 16, 289, and 516 phosphorylated sites identified in *Mycoplasma pneumoniae*, *Streptomyces coelicolor* and *Mycobacterium tuberculosis*, respectively [26, 30, 31]. These findings imply distinct protein phosphorylation patterns in individual species.

In this study we presented a Ser/Thr/Tyr phosphoproteomic analysis of *S*. *meliloti* in the minimum medium at stationary phase. Bacteria at this condition are characterized by their non- or slow-growing status, which is similar to that in soils of limited nutrients and various stresses. A global view of sequence features and functional categories for identified phosphopeptides/phosphoproteins were presented. Moreover, the potential implication of site-specific Ser/Thr/Tyr phosphorylation in cellular physiology was discussed for proteins with known or predicated physiological roles.

## Materials and Methods

### Strain and growth conditions

*S*. *meliloti* CCBAU01290 was isolated from experiment station of Institute of Horticulture, Inner Mongolia Municipality (E117.70, N40.80), China [32, 33]. The medium used for culturing *S*. *meliloti* strains was TY medium, or minimum medium (D-mannitol 10 g, morpholinepropanesulfonic acid 2.09g, K_2_HPO_4_ 1 g, KH_2_PO_4_ 1 g, Na_2_HPO_4_ 0.12g, FeCl_3_ 0.01 g, MgCl_2_ 0.25 g, NH_4_Cl 1 g, CaCl_2_ 0.1 g in 1 liter medium) at 28°C[34].

### Cell culture and lysate preparation for phosphoproteomic analysis

CCBAU 01290 was grown in TY medium. Cell pellets were washed twice with sterilized distilled water and cultured in MM at an initial OD_600_ of 0.1 at 28°C. Two biological replicates were performed in this work. Stationary phase cells were collected by centrifugation at 4000 rpm for 30 min at 4°C and washed twice in sterilized distilled water. The cell pellets were resuspended in 1.5 mL ice-cold L3 lysis buffer (Urea 210 g, Thiourea 76 g, SDS 1 g, Tris 1.2 g, 1mM PMSF (Phenylmethanesulfonyl fluoride), 2mM EDTA, 50mM NaF, 1mM Na_3_VO_4_, 50 mM β-sodium glycerophosphate, 10 mM Na_4_P_2_O_7_.10H_2_O, in 500 mL, pH 8.0), and 10 mM DTT was added 5 min later. Cell wall and membranes were disrupted by sonication (2 s on, 2 s off, electric power at 450 w for 15 min) on ice. The extracts were centrifuged for 15 min (4°C) at 30000 g, and the supernatant was incubated in 56°C water bath with 10 mM DTT added. Then, the sample was treated with 55 mM iodoacetamide in darkness for 45 min. The protein extracts were precipitated with 5 volumes of acetone for 2 h. The sample was air-dried after it was centrifuged for 15 min (4°C) at 25000 g. The air-dry sample was dissolved in 200 μL 0.5M TEAB buffer and sonicated (2 s on, 2 s off, 450 w) for 15 min. The residual cellular debris was removed by centrifugation (30000 g, 4°C, 15 min).

### Protein digestion and phosphopeptide enrichment

The protein sample in 0.5 M TEAB buffer was digested with sequencing grade modified trypsin (Protein:Trypsin = 20:1 w/w) at 37°C for 4 h, and further digested with trypsin at same proportion at 37°C for 8 h. The tryptic digestion peptide mixtures were precleaned with a Strata X C18 column for desalting and dried in a vacuum centrifuge. The dried peptide mixtures were redissolved in 65% acetonitrile (ACN), 2% trifluoroacetic acid (TFA) buffer and saturated in 20 mg/mL glutamate acid buffer. Phosphopeptides in the peptide mixtures were enriched by using Phosphopeptide Enrichment TiO_2_ kit (GLScience, Saitama, Japan), every 1000 μg peptides in mixtures were loaded onto the column with 500 μg equilibrium TiO_2_ phosphobind resin. After 20 min incubation, the phosphopeptides were eluted successively with 65% ACN, 0.5% TFA, and then, 65% ACN, 0.1% TFA, continued with 50% ACN, 1.1% NH_4_OH, followed with 50% ACN, 3% NH_4_OH. Then the enriched phosphopeptides were dried in a vacuum centrifuge for LC-MS/MS analysis.

### LC-MS/MS analysis

Mass spectrometry was performed on Triple TOF 5600 system (AB SCIEX, Concord, ON) coupled to a LC-20AD Nano-HPLC system (SHIMADZU, Japan). For each analysis, the dried peptide fractions were resuspended in buffer A containing 5% ACN and 0.1% formic acid (FA) to a concentration of about 0.5 μg/μL and centrifuged at 20000 g, 4°C for 10 min to remove insoluble substances. Peptide mixtures were loaded onto the trap column with an injection volume of 5 μL and at the flow rate of 8 μL/mL in 4min. The peptide samples were subsequently conducted on the analytical column at the flow rate of 300 nL/min. And then, the peptides were firstly eluted from the column with 5% buffer B (95% ACN, 0.1% FA) for 5 min, followed by elution from the column with linear solvent gradients (35min gradient from 5%-35% buffer B, increased to 60% buffer B in 5min, then increased to 80% in 2 min and held at the gradient for 2 min, back to 5% in 10 min and equilibrated the column at the gradient for 10min. The MS analysis was performed on a Nanospray III source and the instrument was operated in the positive ion mode, with an ion-spray voltage of 2.5 kV and an interface heater temperature of 150°C. MS spectra were acquired in high-resolution mode (>30000) using 100 ms accumulation time per spectrum and from one full MS scan (m/z 300–1500). A maximum of 40 precursors per cycle were chosen for fragmentation from each MS spectrum, 2.8s for each cycle.

### Raw data processing

For Triple TOF 5600 data of phosphopeptides, the peak list of each raw MS data files were generated into mgf format files. All spectra were searched using Mascot v2.3.02 (Matrix Science, London, UK) against *Sinorhizobium meliloti* protein database (http://www.ncbi.nlm.nih.gov/genome/genomes/1004). The search criteria were set as follows: peptide mass tolerance of ± 0.05 Da, fragment mass tolerance of ±0.1 Da, trypsin specificity allowing up to 2 missed cleavages, carbamidomethylation of Cys was set as fixed modification, while oxidation of Met, phosphorylation of serine, threonine and tyrosine were set as variable modifications. The phosphopeptides with expected value below 0.1 were considered as confident matches. Detailed characteristics of unique phosphopeptides identified in two independent experiments are shown in S1 Table. 82 out of 88 phosphopeptides have expected values below 0.05 and six phosphopeptides have the values ranged from 0.051 to 0.061. Localization probability of phosphosites was calculated by using phosphoRS method [35]. Annotated MS/MS spectra are available upon request. Raw data have been deposited to the ProteomeXchange Consortium (http://proteomecentral.proteomexchange.org) via the PRIDE partner repository [36] with the dataset identifier PXD002064.

### Bioinformatic analysis

A 13 mer file (± 6 residues around Ser/Thr) was generated to compare the sequence patterns around phosphorylated or non-phosphorylated Ser/Thr using Weblogo [37], and phosphorylated Tyr was not analyzed due to its low frequency in this study. Secondary structures of all identified phosphopeptides were predicted with a web tool NetSurfP [38]. We compared the mean secondary structure probabilities of identified phosphopeptides with all the identified phosphorylated proteins. The subcellular localization of all identified phosphoproteins were analyzed by using PSORTb program [39]. To understand more about the specificity between eukaryotic and prokaryotic kinases, a web-based tool SCANSITE was used in this study to search for the identified phosphorylation sites with default parameters [40]. The functional information of the identified phosphoproteins was obtained by searching GO (Gene Ontology) and COG (Cluster of Orthologous Groups of proteins) databases [41, 42]. BLASTP was used to check the conservation of phosphoproteins among 20 bacterial species with the information of phosphoproteomes [43]. The combined alignment must include at least 50% of its full length in the alignment and the BLAST hits between the homologs must have a bit score greater or equal to 50, with an E-value < 1e-05.

## Results and Discussion

### Sequence features of phosphoproteome of *S*. *meliloti*

**Fig 1:**
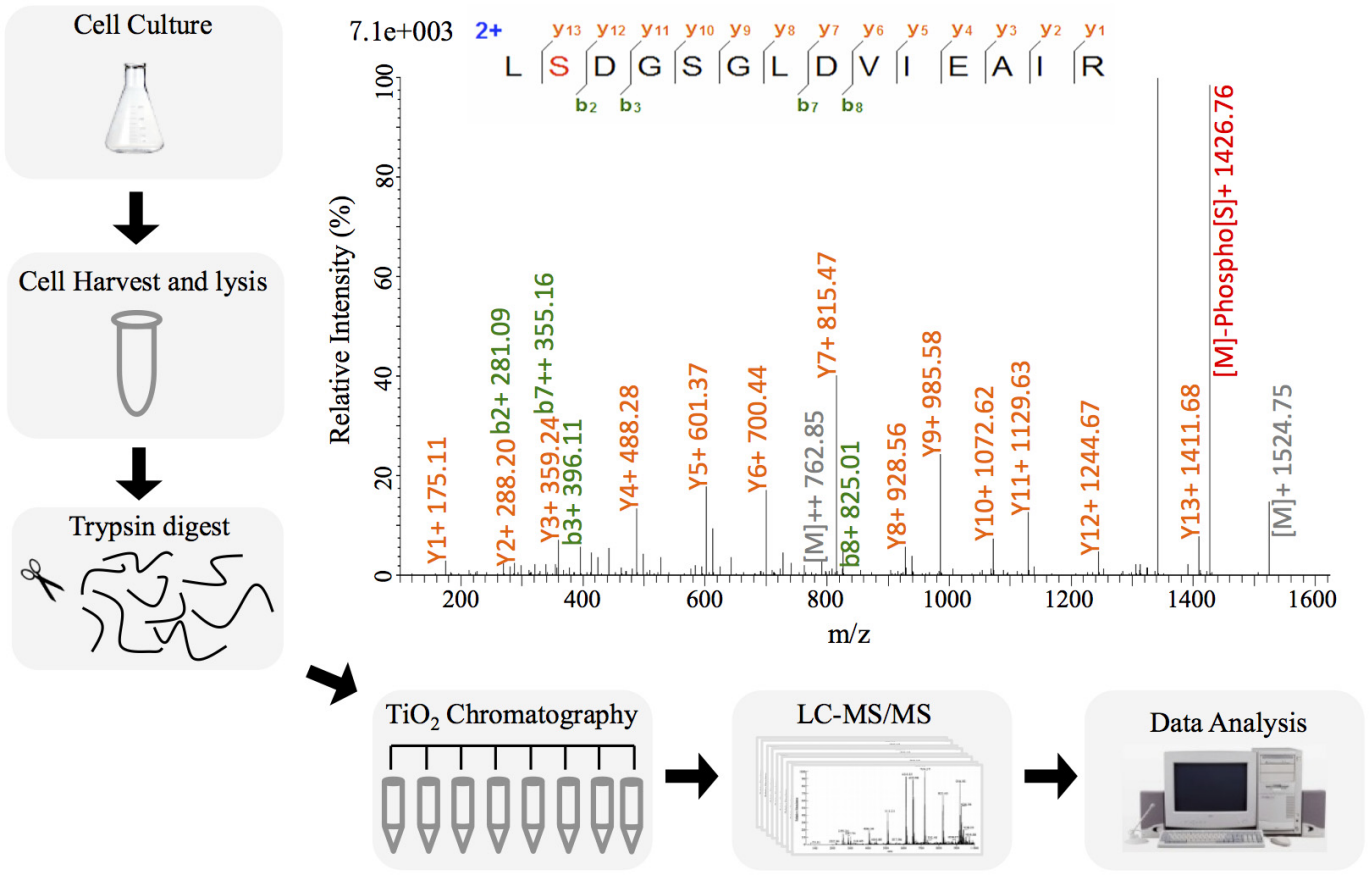
Overview of the workflow and a representative MS/MS spectrum. MS/MS spectrum of the phosphopeptide L(pS)DGSGLDVIEAIR from the Ser-phosphorylated two-component response regulator ActR are shown, and the most abundant fragment ion corresponds to neutral loss of phosphoric acid (98 Da) from the phosphorylated Ser in the intact peptide.

*S*. *meliloti* CCBAU 01290 was found to be an effective microsymbiont, in terms of nitrogen-fixing efficiency and nodule occupation, for major alfalfa cultivars in north China [32, 33], and has been used as rhizobial inoculant in this region. Given the non- or slow-growing feature of rhizobia in natural soils characterized by limited nutrients and various stresses [44], stationary-phase bacterial cells in MM were used to determine the phosphoproteome of *S*. *meliloti* (Fig 1). Within detected 88 unique phosphopeptides, there are 68 phosphosites with high-confidence identification (localization probability above 0.75), 16 being medium-confidence (P value between 0.75 and 0.50), and 12 low-confidence sites (P value below 0.50). Details of these phosphopeptides are listed in S1 and S2 Tables, and a typical annotated spectrum is shown in Fig 1. Among the 96 unique phosphorylated sites in 77 proteins (S2 Table), the distribution of phosphoserine (pS), phosphothreonine (pT) and phosphotyrosine (pY) is 65.6%, 29.2% and 5.2%, respectively (S2 Table). The relative frequencies of these pS/pT/pY in *S*. *meliloti* are similar to *L*. *monocytogenes*, *B*. *subtilis* and *E*. *coli* etc. [23, 24, 27], but distinct from *Campylobacter jejuni*, *Streptomyces coelicolor*, *Clostridium acetobutylicum*, *Latococcus lactis* and *C*. *synechococcus*, which have more pT or a comparable number of pT than pS [28, 45–48].

**Fig 2:**
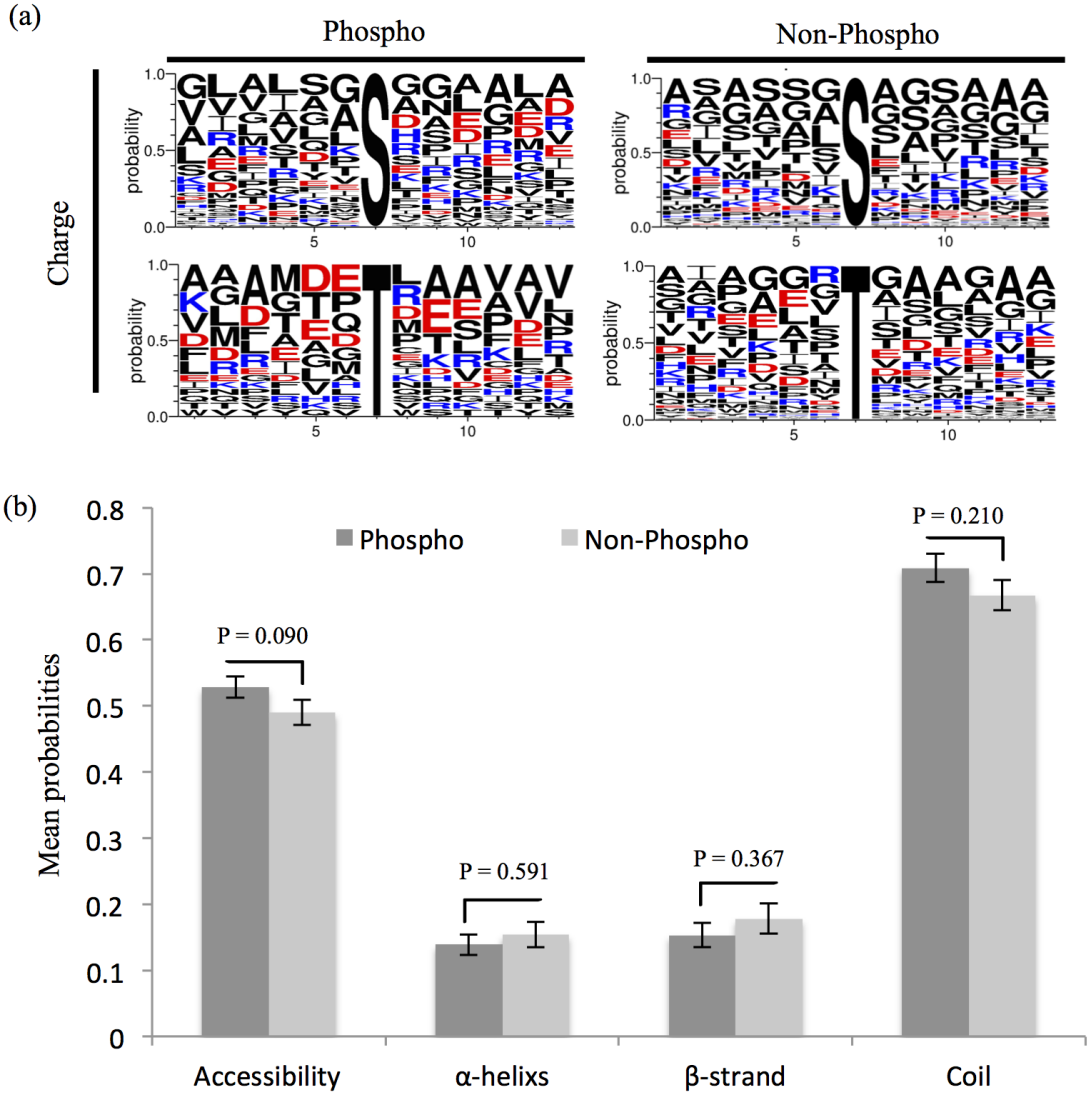
Bioinformatics analysis of phosphorylation sites. (a) Relative abundances of amino acids flanking pS/pT or non-phosphorylated Ser/Thr. Phospho-13-mers and non-phospho-13-mers (6 amino acids upstream and downstream of the phosphorylated or non-phosphorylated site) are shown. Amino acids are colored according to their charge scale. (b) Phosphorylated and non-phosphorylated Ser/Thr/Tyr in protein secondary structures. P-values based on T-test are indicated.

**Fig 3:**
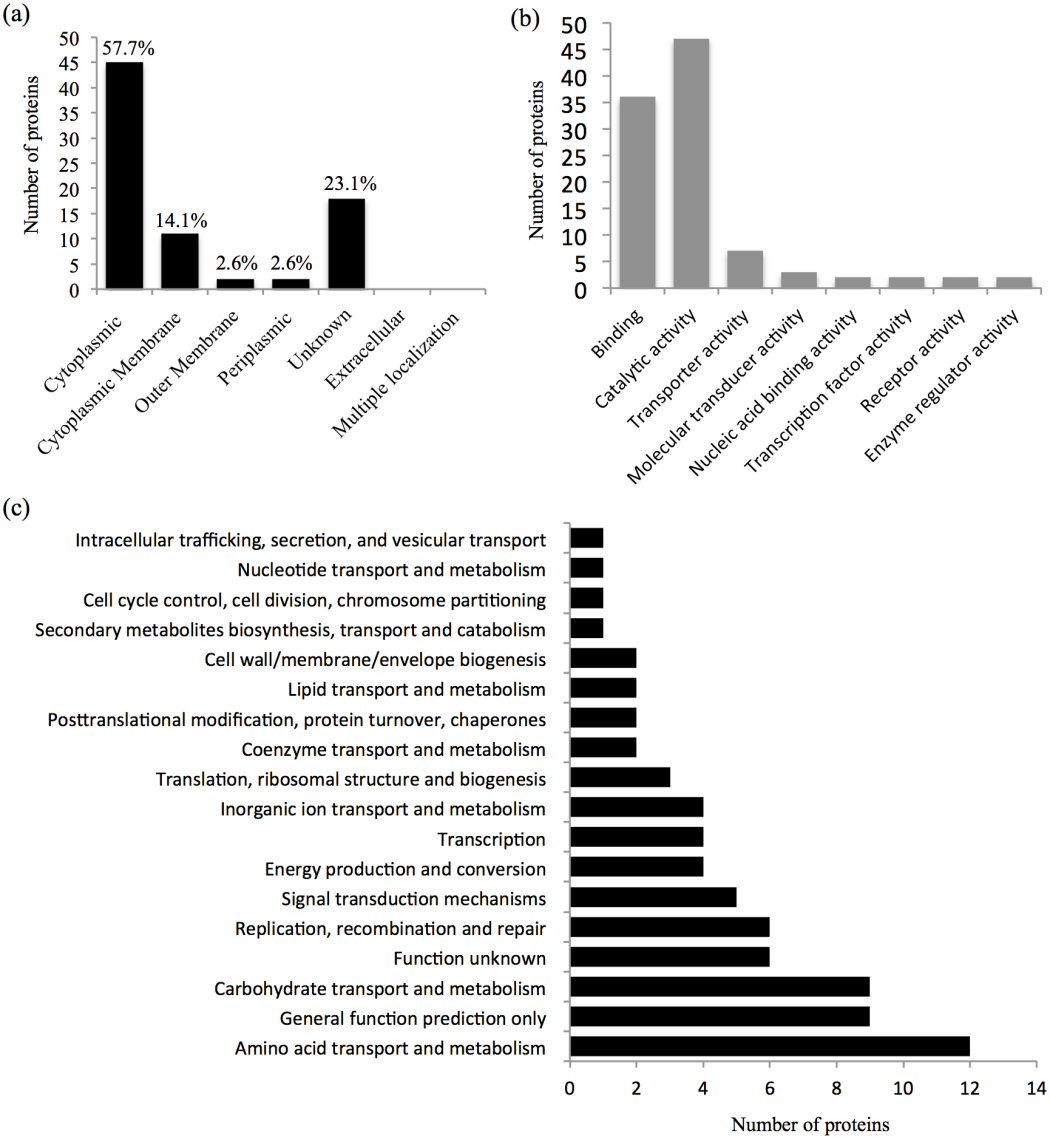
Distribution of identified phosphoproteins. (a) cellular localization, (b) molecular function in gene otology, and (c) cluster of orthologous groups.

To visualize potential preferred sequence pattern around pS/pT in *S*. *meliloti*, relative abundances of amino acids flanking pS/pT or non-phosphorylated Ser/Thr identified in this study were compared. As shown in Fig 2a, neutral amino acids including Ala, Gly, Ser etc. dominate those downstream sites of non-phosphorylated Ser/Thr, whereas the frequency of charged amino acids such as Asp, Glu, and Arg increases in downstream sites of pS and pT. In line with this pattern, the observed phosphorylation sites showed a slightly increased probability of being located on the protein surface than non-phosphorylated sites (Fig 2b, 52.8% versus 49.0%, T-test, P-value = 0.09) as determined by using NetSurfP [38]. Similar trend in solvent accessibility of identified phosphorylation sites was reported in *Phaeodactylum tricornutum* [49]. Moreover, our results also showed that pS/pT is more frequently present in unstructured coil region as demonstrated earlier in other bacteria [28, 50, 51]. Among the identified phosphoproteins, cellular localization for 60/77 proteins could be predicated by using pSORTb [39]. 45 and 11 phosphoproteins are cytoplasmic and cytoplasmic membrane proteins, respectively, whereas four phosphoproteins were predicted as outer-membrane or periplasmic proteins (Fig 3a). As shown in S3 Table, at a stringency of 5%, 18 phosphopeptides matches the target motifs of eukaryotic kinases such as Casein kinase -1 and -2, GSK3b, PLK1, PKC mu, PKC delta, Calmodulin dependent kinase 2, PIP3-binding PH, Erk1 kinase, SH3 etc. This implies potential diverse sources of protein phosphorylation in *S*. *meliloti* as reported in other bacteria [28].

### Global view of phosphoprotein function in *S*. *meliloti*

As shown in Fig 3b, 46.5%, 35.6% or 6.9% of identified phosphoproteins have the molecular function of catalytic, binding or transporter activity, respectively. Other molecular functions include enzyme regulator activity (GlnB and ExoP), molecular transducer activity (two-component response regulators ActR and SMa0114, sensor histidine kinase SMb21546), receptor activity (SMb21546 and FhuA), nucleic acid binding activity (SMc01636 and SMc01406), and transcription factor activity (SMc01636 and SMc01406). To get further insight into the functional categories harbored by the *S*. *meliloti* phosphoproteome, the distribution of phosphoproteins regarding to COG functional categories were investigated (Fig 3c). The average distribution frequency is 6.25% among the 16 categories with known functions. The COG categories with a frequency higher than this average value include transport and metabolism of amino acid (20.68%) and carbohydrate (15.52%), replication, recombination and repair (10.34%), signal transduction mechanisms (8.62%), energy production and conversion (6.90%), transcription (6.90%), inorganic ion transport and metabolism (6.90%). These findings imply that Ser/Thr/Tyr phosphorylation plays an important role in globally modulating rhizobial physiology as revealed in other bacteria [17].

### Phosphoproteins involved in DNA replication, transcription, translation and posttranslational modification

DNA polymerase III subunit beta DnaN (EC:2.7.7.7, pS191 and pS193), double-strand break repair protein AddB (pT47), a histone-like protein HupB (pS51 and pS53), a putative integrase/recombinase SMa2285 (pS36 and pS39), and two putative transposases were identified as Ser/Thr phosphoproteins. Among them, homologs of DnaN and HupB are widely distributed in bacteria (S2 Table). The phosphopeptides LVGFGNF(pS)VSR and LVGFGNFSV(pS)R correspond well to one of the two conserved regions of HupB among diverse bacteria [52], indicating potential regulation of HupB activity by Ser phosphorylation.

Two-component system including a histidine kinase and a response regulator plays a crucial role in regulating the transcription of genes responding to diverse environmental stimuli [17]. Here we identified two cytoplasmic-membrane histidine kinases SMb21546 (pS45) and SMb21209 (pS7), two response regulators SMa0114 (pS87) and ActR (pS77 and pS80) as Ser phosphoproteins. ActS-ActR is a conserved two-component signal transduction system. ActR is involved in regulating CO_2_ fixation, nitrate assimilation and is required for the induction of nitrogen fixation regulators FixK and NifA under low pH and microaerobic conditions in *Sinorhizobium medicae* WSM419 [15]. *SMa0114* in-frame deletion mutants overproduce the carbon storage compound polyhyrdroxybutyrate (PHB), though the transcription of *phb* genes was not affected [14]. Notably, SMa0114 lacks a DNA-binding domain suggesting that SMa0114 functions through protein-protein interactions. The phosphopeptide NVPFIFATGYG(pS)KGLDTR identified herein corresponds to the β4-α4 loop of SMa0114 and similar response regulators, containing the common motif (PFxFA[T/S]GY) [14, 53]. Therefore it is very likely that phosphorylation in the β4-α4 loop could impact SMa0114 conformation and consequently target binding activity [54]. In addition to two-component systems, several transcriptional factors of GntR, AraC and BolA family etc., with different conservation levels, were also identified as phosphoproteins in *S*. *meliloti* (S2 Table). This suggests Ser/Thr phosphorylation of regulator proteins as a common strategy in modulating the transcription process.

Phosphoproteins involved in translation or posttranslational modification include translation initiation factor InfB (pS112), elongation factor Tsf (pS295), a putative amidase SMc02881 (EC: 3.5.1.4, pS179), a putative glutathione S-transferase (EC: 2.5.1.18, pS12) [55] and chaperonin GroEL2 (pS340 and pT185) [56]. All of them are conserved among diverse bacteria (S2 Table). GroEL2 together with GroEL1 are essential for viability of *S*. *meliloti* and overexpression of GroEL2 could complement the symbiotic defect of the *groEL1* mutant [57].

### Phosphoproteins involved in amino acid transport and metabolism

Asd (aspartate-semialdehyde dehydrogenase, EC:1.2.1.11, pS96) catalyzes the reaction from 4-phosphate-L-aspartate to L-aspartate 4-semialdehyde, which is an early branch point in the biosynthesis of methionine and threonine via homoserine, and lysine via meso-2,6-diaminopimelate [58]. Since meso-2,6-diaminopimelate is also a cross-linking agent in bacterial cell walls, Asd is considered as an attractive target for antibiotics [58]. HisB (imidazoleglycerol-phosphate dehydratase, EC:4.2.1.19, pS99 and pT109) and HisC4 (histidinol-phosphate aminotransferase, EC:2.6.1.9, pS369) catalyze the sixth and seventh steps in the pathway forming histidine [59]. MetZ (O-succinylhomoserine sulfhydrylase, EC:2.5.1.48, pS112) is involved in cysteine and methionine biosynthesis from homoserine [60]. In addition to these cytoplasmic phosphoproteins, six proteins on cytoplasmic membrane were also found, such as AapP (broad-specificity amino acid transporter ATP-binding protein, pT181) [61], OppD (required for uptake of tetrapeptides and certain tripeptides, pT54) [62] and nitrogen regulation protein GlnB (pY51) [63].

### Phosphoproteins involved in carbohydrate transport and metabolism, energy production and conversion

**Fig 4:**
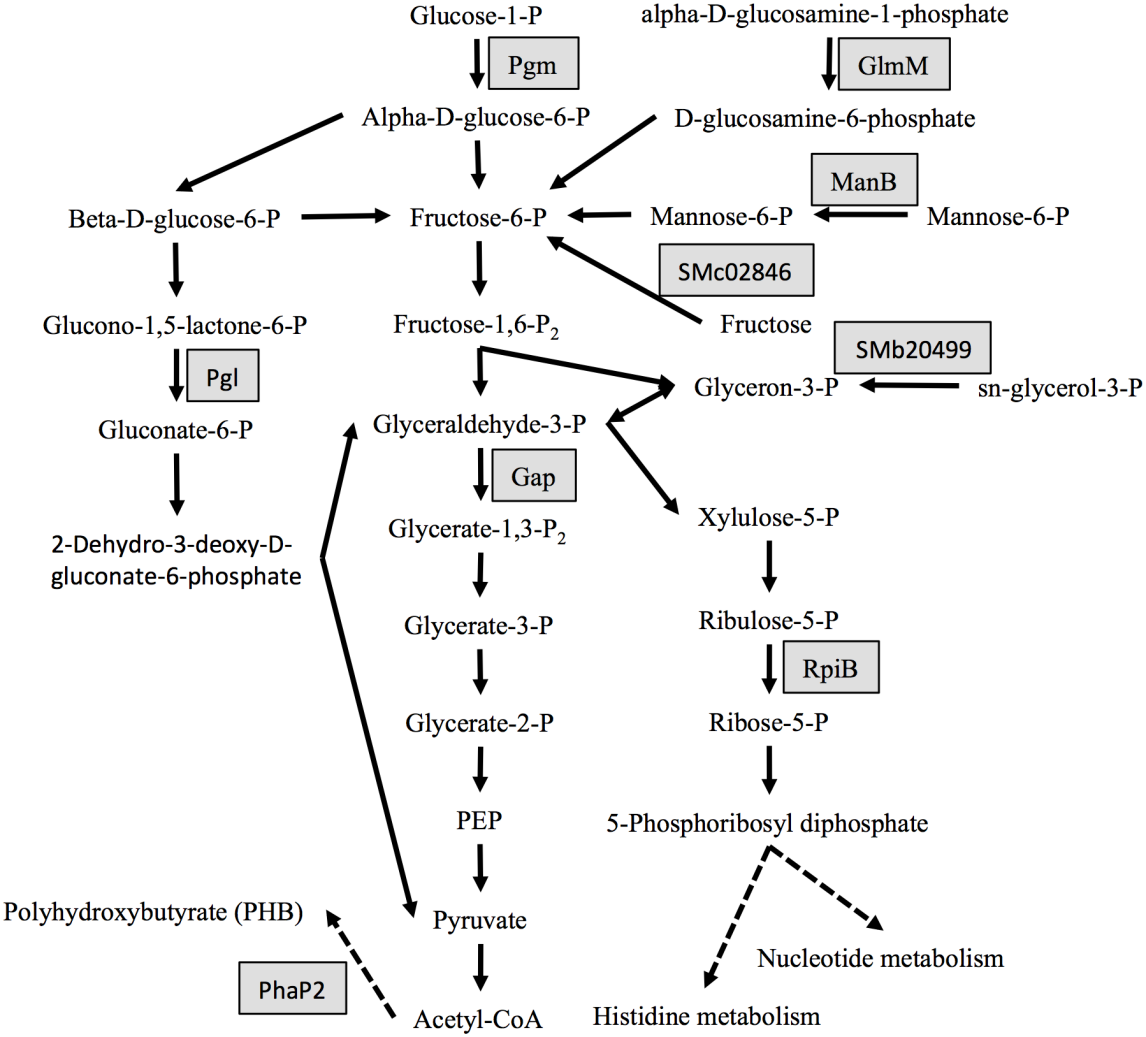
Phosphoproteins identified in carbohydrate metabolism. Gray box indicates phosphoproteins. PEP, phosphoenolpyruvate.

As shown in Fig 4, Pgm (phosphoglucomutase, EC:5.4.2.2, pS112) catalyzes the reaction from alpha-D-glucose-1-phosphate to D-glucose-6-phosphate, which could be further converted to fructose-6-P by Pgi (glucose-6-phosphate isomerase) in the glycolysis pathway. Another phosphorylated glycolytic protein is Gap (glyceraldehyde-3-phosphate dehydrogenase, EC:1.2.1.12, pT210, pS211), which dehydrogenates glyceraldehyde-3-phosphate and adds an inorganic phosphate forming 1,3-bisphosphoglycerate. Moreover, Pgl (6-phosphogluconolactoase, EC:3.1.1.31, pT45 and pT136) and RpiB (ribose 5-phosphate isomerase B, EC:5.3.1.6, pS95) in pentose phosphate pathway, GlmM (phosphoglucosamine mutase, EC:5.4.2.10, pS102) in amino sugar metabolism, ManB (phosphomannomutase, EC:5.4.2.8, pS99) in mannose metabolism, and SMc02846 (D-fructokinase, EC:2.7.1.4, pS198) in fructose metabolism were also phosphorylated at Ser and/or Thr. Certain phosphosites in these proteins with key cellular function were also reported in other bacteria [23, 24, 27]. For example, AAALSMIPT(pS)TGAAK in *S*. *meliloti* Gap is similar to those of *L*. *lactis* and *L*. *monocytogenes* [27]. GlmM is phosphorylated at S102 (ADIGVMISA(pS)HNAFR) in *S*. *meliloti*, and *E*. *coli* GlmM with the same conserved pS102 is essential in cell wall peptidoglycan and lipopolysaccharide biosynthesis [64, 65]. Notably, two phosphorylated periplasmic proteins were also identified herein, a putative dicarboxylate transporter (SMb21438, pS9) and a putative sugar transporter SupA (SMb20484, pT27). The expression of SupA could be induced by potassium and is essential for the adaptation of *S*. *meliloti* to the high level of potassium [66]. PHB granules assist bacterial survival under nutrient-fluctuating conditions. PhaP2, one of the two major phasins (PHB granule-associated proteins) involved in regulating PHB synthesis and granule formation in *S*. *meliloti* [67], was found to be phosphorylated at Ser40 and Ser43.

### Phosphoproteins in transport and metabolism of inorganic ion, lipid, coenzyme, nucleotide, and exopolysaccharide

CysQ (EC: 3.1.3.7, pT87) could prevent accumulation of 3’-phosphoadenoside 5’-phosphosulfate (PAPS), the toxic intermediate in the conversion of sulfate into sulfite [68, 69]. FhuA is a specific ferrichrome outer-membrane receptor and has three TonB box regions [70]. Here we find a phosphopeptide GPTALL(pY)GISPNSSVGGSINIVPKR corresponding to the TonB box III of FhuA, implying potential regulation of ferrichrome utilization by Tyr phosphorylation in *S*. *meliloti*. AccD (acetyl-CoA carboxylase, EC:6.4.1.2, pT238) and FabG (3-oxoacyl-acyl-carrier protein reductase, EC:1.1.1.100, pS15) are involved in fatty acid biosynthesis. Adk (adenylate kinase, EC:2.7.4.3, pS30) in purine metabolism, NadD (nicotinic acid mononucleotide adenylyltransferase, EC:2.7.7.18, pT165) in nicotinate and nicotinamide metabolism, PanB (3-methyl-2-oxobutanoate hydroxymethyltransferase, EC:2.1.2.11, pS256) in pantothenate and CoA biosynthesis were also found to be phosphorylated. In *S*. *meliloti*, ExoP is described as an autophosphorylating protein tyrosine kinase and is involved in polymerization and secretion of succinoglycan [22, 71], which is required for successful invasion of alfalfa nodules [72]. In this study, phosphorylated Y774 and Y775 of ExoP were identified. In line with the finding herein, ExoP.Y775S resulted in a significant decrease in tyrosine phosphorylation detected by the anti-phosphotyrosine antibody PT-66 [22].

## Conclusions

**Fig 5:**
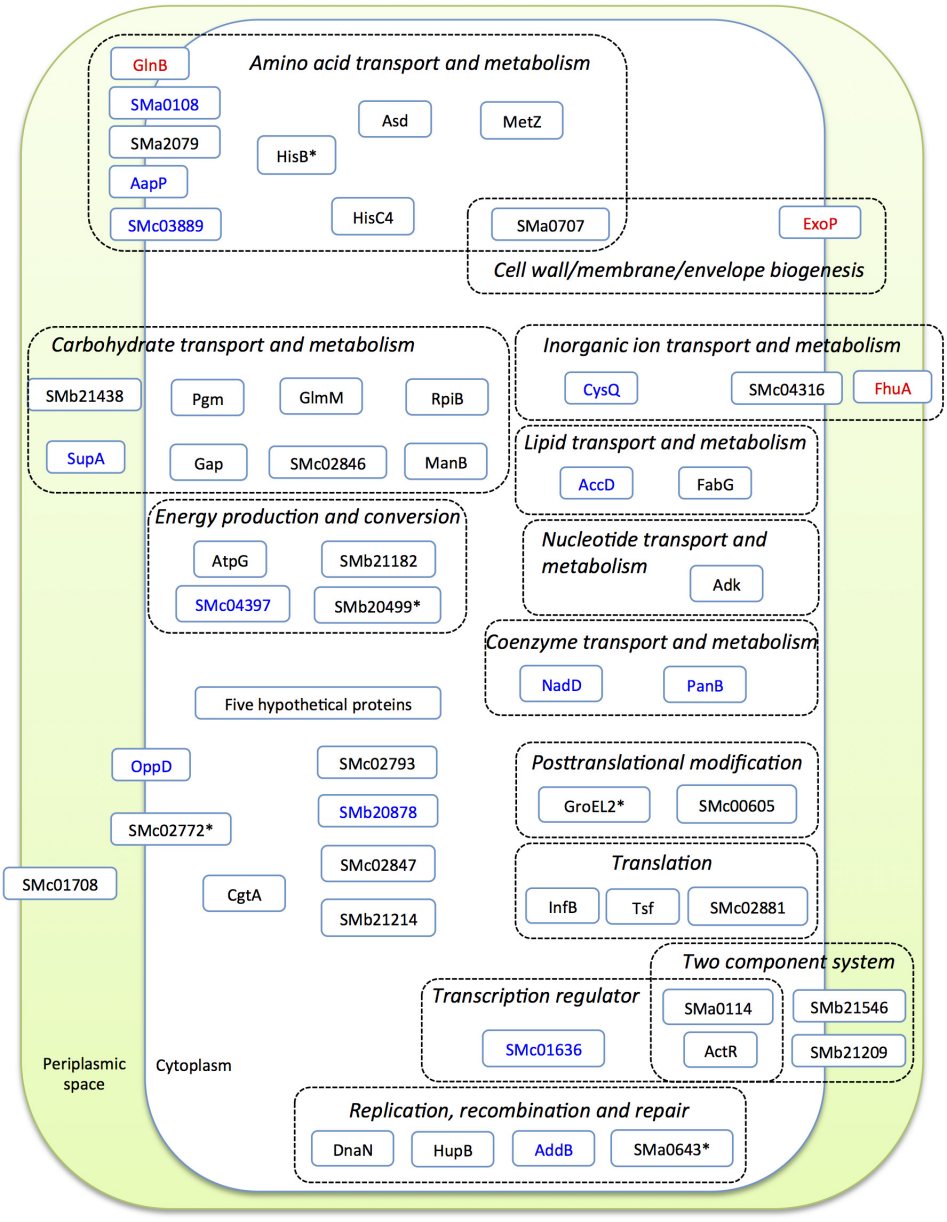
Global view of identified phosphoproteins with predicated cellular localization. The cluster of orthologous group is shown. Proteins phosphorylated at Ser (black), Thr (blue), or Tyr (red) are presented in different colors; * indicates phosphorylated Thr is also identified in this protein.

Rhizobia live saprophytically in soil and occasionally form nitrogen-fixing nodules on legumes. In this study we carried out a site-specific phosphoproteomic analysis of the alfalfa microsymbiont *S*. *meliloti* growing in minimum medium at stationary phase. Ser/Thr/Tyr phosphorylation sites were identified in proteins of diverse functions regarding to COG categories (Fig 5). This not only confirmed some conserved phosphosites reported in homologous proteins of other bacteria but also revealed some previously unknown phosphorylation sites in conserved motif of proteins with important physiological roles. Some of these proteins have been demonstrated as essential elements in rhizobial adaptations to diverse stress conditions, though a considerable number of phosphoproteins have not been studied yet. Therefore, the site-specific phosphoproteome of *S*. *meliloti* obtained herein could provide valuable information for designing future experiments that address the molecular mechanism of related proteins involved in rhizobial adaptation to different environmental conditions.

## Supporting Information

S1 Table Detailed characteristics of identified phosphopeptides. (XLSX)

S2 Table Unique phosphopeptides identified in this study. (XLSX)

S3 Table SCANSITE prediction for binding motifs of eukaryotic protein kinases at high (0.2%), medium (1.0%) and low stringency (5.0%) within identified phosphorylated sites of *Sinorhizobium meliloti* CCBAU 01290. (XLSX)
